# Patience and its relationship to stress tolerance in relation to demographic factors of the medical system in Bethlehem Governorate during the COVID-19 pandemic

**DOI:** 10.3389/fpsyg.2023.1059589

**Published:** 2023-02-02

**Authors:** Nahida Saba Al-Arja

**Affiliations:** Department of Social Sciences, Faculty of Arts, Bethlehem University, Bethlehem, Palestine

**Keywords:** patience, stress tolerance, medical system, COVID-19 pandemic, Palestine

## Abstract

**Objective:**

The present study aims to identify the relationship between patience and stress tolerance in the medical system in Bethlehem Governorate during the COVID-19 pandemic, as well as to identify the impact of several demographic variables on it.

**Methods:**

A random sample of 160 members/workers of the medical staff completed the Patience Scale and Coping Processes Scale questionnaire.

**Results:**

It showed there is a significant positive effect for patience on stress tolerance and there were statistically significant differences in the level of patience in favor of males and single workers. It was also found that there is no difference in the specific duties of a health care worker and no variance of statistical evidence was found in the level of patience due to work with COVID patients but there were differences in stress tolerance in favor of those who do not work with COVID patients. It was also found that there is a significance in the level of stress tolerance in favor of the National Center in regard to bearing pressure. The study findings indicate there was a negative correlation with statistical significance between stress tolerance and age. The nature of stress changes with age, from episodic to chronic, which in turn affects appraisal and coping processes.

**Conclusion:**

This study, which was conducted on a sample of Palestinian medical workers in the Bethlehem area, showed that at the beginning of the pandemic, the medical system in Palestine was not ready to handle COVID-19, and had no precautions to prevent the disease. In spite of that, the doctors and nurses who were undergoing psychological pressure were able to stand at the front line and face the outbreak of the coronavirus.

## Introduction

From the city of Wuhan, the capital of Hubei province, China, and late in 2019, the new coronavirus “COVID-19” started to spread (to the rest of the world). Many believed that they were safe from the virus. Yet in record time it swept all parts of the earth. The world was not prepared at all to confront it and so humanity was completely devastated. Everyone was confused. The shock was intense and left a great impact on individuals, families, societies, and whole nations. The first days of the pandemic, left humanity dumbfounded, unable to absorb the shock repercussions and alienated all from their previous routine lives (Al-Fiqi and Abul-Fotouh, 2020).

Palestine was no exception. It struggled nervously as the first cases of COVID-19 were discovered among some international tourists in Bethlehem. Then the virus appeared in all other directorates.

The measures undertaken by the (PNA) to stop or limit the spread of the virus, included: a general lockup, social distancing, restriction of movement, and closure of schools and universities. Work in almost all sectors stopped and people were required to wear masks whenever they were in public, and a state of emergency was declared by the Palestinian Authority (Abdel Hamid, 2020).

In contrast, the impact of the measures imposed by governments to fight the spread of COVID-19 across the different countries around the world on the mental health, economics, and well-being was great, was enormous. Some people likened this effect to a world war, except that “the whole world was standing in the same trench.” Scientists in the major research centers in the world worked around the clock to learn more about this virus and to develop tools to combat it. Attempts to create a vaccine to treat COVID-19 finally bore fruit and the damage caused by the virus was finally brought under control (Abdel Hamid, 2020).

I must also add the developments that took place in remote psychotherapy in Palestine and elsewhere (Abdel Hamid, 2020).

The present research was designed to broaden our understanding of the relationship between stress tolerance in the medical system in Bethlehem Governorate during the COVID-19 pandemic, by examining the potential effect of patience. That is if patience has a relationship with enduring stress among medical staff while working with a COVID-19 patient.

The fast-paced world we live in makes us impulsive, stressed, and sad most of the time. Yet with patience, we can endow the world with a unique edge. This is not an easy thing, as the world is in a dire need for each of us to contribute our best. With patience, we can live in harmony with the natural rhythm of life because it enables us to deal with life (Ryan, 2003).

Patience is a desirable quality, but when a culture is based on the mere satisfaction of needs, this quality has become a luxury. Getting what we want when we want it weakens one of our most important characteristics, namely the ability to be patient (Cunningham, 2011).

Patience provides us with persistence to achieve our goals and dreams, bestows on us a feeling of inner tranquility, and helps us to put things in their proper perspective. Instead of being hopeless, and driving others away from us, we become a source of happiness and comfort for them. Patience makes us more confident in our ability to face what may come along our path. Patience includes self-control (Ryan, 2003).

Patience is very important to us as human beings, especially during an age which is full of contradictions, problems, wars, and the spread of a pandemic (COVID-19). The sources of psychological crisis have multiplied rapidly and new coping strategies have thus become imperative. Al-Esawy (1988) believes that, from a psychological point of view, patience is a widespread feature that covers all aspects of human life and forms a basis of his mental, psychological, and motor behavior.

Patience is defined as the way a person endures/withstands frustration, adversity, or suffering when there is no choice but simply to wait (Schnitker, 2012).

Paulson (2010) describes as patient every person who has faced or is constantly facing a difficult time, is waiting for a life-changing medical report, or has heard news of a collapse in the stock market, or is fired from his job, or has lost or is about to lose a loved one. Optimism, though may seem otherwise, can have a powerful impact on one’s life, personally or professionally.

Ryan (2003) emphasized that patience gives us the ability to withstand obstacles and troubles that we face in our lives, such as failure at work, disappointment, serious disability, or material disasters with forbearance and acceptance and to respond to life’s pressures and tensions with courage, strength, and optimism because it is an integral part of life.

Many researchers believe that we are in an era of constant stress and psychological crises. This requires each of us to exert his utmost to restore one’s harmony with himself and with the environment in which he lives (Al-Zghoul and Abdel-Fatthah, 2003).

Using their cognitive theory, Lazarus and Delongis (1983) defined psychological stress as a state of emotional tension arising from a situation in which there is a disturbance in the physiological functions and insufficient cognitive functions which are necessary for a particular situation. It appears that when an individual is exposed to a stressful situation, his response takes the form of an internal behavior to defend against emotional excitement, tension, and anxiety, storing the lost energy and reaching a state of psychological equilibrium through certain strategies known as coping processes. If these processes are inappropriate, the resistance becomes ineffective, and the individual reaches a stage of psychological exhaustion. Over time, the resistance weakens and the individual reaches what Sely (1980) calls *diseases of compatibility*. In a stressful situation, the physical and psychological organs of the individual work like an alarm clock that does not stop ringing until after it has exhausted its energy.

Due to their nature, some professions cause more tension than others. For example, in the health care field, workers deal directly with people and devote themselves to serving patients and helping them recover their health and rehabilitate them so as to rely on themselves. These professionals are clearly subject to more pressure than workers in other fields. The burdens associated with their work and the great effort they put in while dealing with patients are enormous, not to mention, the psychological pressure they face while performing their various duties (Tantoui, 2020).

The infections that doctors and health workers may be exposed to are one of the most serious health hazards that are not present in most other sectors. The reasons are endless; they work among patients and may transmit disease to other patients and when they go home, they may transmit infections to their families. More importantly, hospital infections are often characterized by their resistance to antibiotics. Health care professionals may themselves fall victims to new diseases which may not have a cure yet. This makes protecting health care workers from infection an absolute necessity (Tantoui, 2020).

Since the beginning of the COVID-19 pandemic in March 2020, health care professionals, including nurses and doctors, have been involved in a war against this virus. In Palestine, this war has so far claimed the lives of 17 medical staff members, 11 from the West Bank, and 6 from the Gaza Strip, while they were performing their professional duty (Al-Rimawi, 2021).

Research indicates that since the spread of the coronavirus, the medical system has been facing an enormous amount of pressure that makes it imperative for health care professionals to be patient and to acquire positive coping techniques to combat psychological stress. van Roekel et al. (2021) have shown that health care workers who work with COVID-19 patients are more physically exhausted and need mental and physical support to survive the pandemic.

Javed et al. (2020) show that doctors, nurses, and paramedics working as a front-line force to fight the COVID-19 outbreak are more susceptible to develop mental health symptoms. Fear of catching a disease, long working hours, unavailability of protective gear and supplies, patient load, unavailability of effective COVID-19 medications, death of their colleagues after exposure to COVID-19, social distancing and isolation from their families and friends, and the dire situation of their patients almost certainly have a negative effect on the mental health of workers in the health sector.

Vindrola-Padros et al. (2020) also show that the positive aspects of daily work reported by HCWs included solidarity between colleagues, the establishment of well-being support structures and a feeling of being valued by society.

To the best of our knowledge, very little research has been conducted on patience and its relationship to Stress Tolerance among health care professionals in the medical system in Bethlehem Governorate during the COVID-19 pandemic. It is hoped that the present research will fill this research gap.

This study, therefore, aims to identify the relationship between patience and Stress Tolerance in the medical system in Bethlehem Governorate during the COVID-19 pandemic, as well as to identify the impact of several demographic variables on it.

## Materials and methods

### Sample selection and procedure

Random sample was used to select doctors and nurses, the researcher administered questionnaires to the doctors and nurses in the hospitals. Ethics approval for the study was obtained from the Palestinian Ministry of Health.

### Participants and procedures

The study sample consisted of 160 members/workers of the medical staff in government hospitals in Bethlehem Governorate, Al-Hussein Hospital, Dr. Muhammad Saeed Kamal Hospital–Bethlehem Hospital for Psychiatric and Neurological Diseases, National Center for COVID-19 treatment, Health Directorate in Bethlehem from October to December 2020.

### Measures

The questionnaire battery, including a consent form, a demographic information sheet, and the Arab version of the patience scale and coping processes scale was administered.

#### The patience scale

The Patience Scale (Arnaut, 2016) contains 27 items rated on a 6-point Likert–type format anchored by 1: No and 6: Very much. Cronbach’s Alpha reliability (0.77) denoting internal consistency and temporal stability.

#### Coping processes scale

Coping Processes Scale (Ibrahim, 1994) contains 42 items, rated on 4-point Likert-type format, anchored by 1: very much and 4: no. Cronbach’s Alpha reliability of Coping Processes among medical staff was (0.73) denoting good internal consistency and temporal stability.

The descriptive analytical method was adopted in this study in order to answer the questions and assumptions presented by the study.

### Statistical analysis

Descriptive methods, data analysis sample size calculations, the frequencies, the standard deviations, the percentages of variance, the *t*-test and Scheffe test were used to verify the sources of variances were performed using the statistical package for social sciences software SSPS version 16.

## Results

### Patience and stress tolerance

The findings of the present study of the total sample of participants (*n* = 160), simple linear regression show that there is a positive effect at a significant level for patience on stress tolerance among medical staff in Bethlehem governorate. The correlation coefficient was (0.478), *T* value signification level was (2.87), and was statically significant (0.01) (Table 1).

**TABLE 1:** Results of simple linear regression to examine the effect of patience on stress tolerance.

Correlation coefficient	Inflection coefficient	Slope of the regression line	*T*-value signification level	Statistical significance
0.478	0.228	0.137	2.87	0.01

### Patience, stress tolerance, and gender

Table 2 shows the results of *t*-test for variance in total means of the level of patience and stress tolerance. It is clear that there were statistically significant differences at the level at the level (α = 0.05) level patience in favor of males, and there were no differences in stress tolerance due to the gender variable. The level of significance was (0.023). The mean was (100.04) for males and (96.91) for females; a relatively close percentage of the gender (Table 2).

**TABLE 2:** Results of *t*-test for variance in total means of the level of patience and stress tolerance, due to gender.

	Gender	*N*	Mean	Std.	*T*-value	Degree of freedom	Level of significance
Patience	Male	52	100.04	7.68	2.301	158	0.023
	Female	108	96.91	8.23			
Stress tolerance	Male	52	110.00	12.39	0.209	158	0.834
	Female	108	109.59	11.10			

### Patience, stress tolerance, and social status

There were statistically significant differences in the level of patience at the level (α = 0.05) in favor of single staff members, and there were no differences in stress tolerance due to the social status variable; the level of significance was (0.006). The mean for single members was (100.86) and (96.88) for married staff (Table 3).

**TABLE 3:** Results of *t*-test for variance in total means of the level of patience and stress tolerance, due to social status.

	Social status	*N*	Mean	Std.	*T*-value	Degree of freedom	Level of significance
Patience	Single	42	100.86	10.59	2.765	158	0.006
	Married	118	96.88	6.87			
Stress tolerance	Single	42	110.90	13.38	0.773	158	0.440
	Married	118	109.31	10.78			

### Patience, stress tolerance, and exposure to COVID-19 infection

It is clear that there were no statistically significant differences in the total degree averages of the at the level (α = 0.05) of patience and stress tolerance, due to COVID-19 infection; the level of significance was (928) (Table 4).

**TABLE 4:** Results of *t*-test for variance in total means of level of patience and stress tolerance, due to exposure to COVID-19 infection.

	Have you been infected with corona?	*N*	Mean	Std.	*T*-value	Degree of freedom	Level of significance
Patience	Yes	44	97.95	9.38	0.028	158	0.978
	No	116	97.91	7.70			
Stress tolerance	Yes	44	109.59	10.86	−0.091	158	0.928
	No	116	109.78	11.77			

### Patience, stress tolerance, and working/contact with COVID patients?

It is clear that there were no statistically significant differences in the level of patience due to work with COVID patients and there were statistically significant differences at the level (α = 0.05) level in stress tolerance in favor of those who do not work with COVID patients. The level of significance was (0.000). The mean of those who do not work with COVID patients was (112.33) and who work with COVID patients was (105.14); this is a relatively close result (Table 5).

**TABLE 5:** Results of *t*-test for variance in total means of the level of patience and stress tolerance, due to working with COVID patients.

	Do you work with COVID patients	*N*	Mean	Std.	*T*-value	Degree of freedom	Level of significance
Patience	Yes	58	97.07	7.11	−1.000	158	0.319
	No	102	98.41	8.71			
Stress tolerance	Yes	58	105.14	11.75	−3.979	158	0.000
	No	102	112.33	10.55			

### Patience, stress tolerance, and profession

There were no statistically significant differences in the total degree averages of the level of patience and stress tolerance, due to the profession variable, the level of patience significance was (0.687) and level of stress tolerance was (0.315) (Table 6).

**TABLE 6:** One way ANOVA analysis of the differences in the degree of patience and stress tolerance, due to profession.

Dimension	Source	Sum of squares	Degree of freedom	Squares mean	*F*-value	Level of significance
Patience	Between groups	50.633	2	25.317	0.376	0.687
	Inside groups	10558.467	157	67.251		
	Total	10609.100	159			
Stress tolerance	Between groups	307.408	2	153.704	1.165	0.315
	Inside groups	20708.492	157	131.901		
	Total	21015.900	159			

### Patience, stress tolerance, and the workplace

There were statistically significant differences in the level of stress tolerance, but there were no statistically significant differences at the level (α = 0.05) in the degree of patience. One Way ANOVA test was used to identify these differences showed differences between the Bethlehem Health Directorate and the National Center were in favor of the National Center with a mean of (99.52) and a significance level of (0.000) (Table 7).

**TABLE 7:** One way ANOVA analysis of the differences in degree of patience and stress tolerance due to workplace.

Dimension	Source	Sum of squares	Degree of freedom	Squares mean	*F*-value	Level of significance
Patience	Between groups	121.093	2	60.547	0.906	0.406
	Inside/within groups	10488.007	157	66.803		
	Total	10609.100	159			
Stress tolerance	Between groups	837.434	2	418.717	3.258	0.041
	Inside/within groups	20178.466	157	128.525		
	Total	21015.900	159			

### Patience, stress tolerance, and age

It is clear that there was a negative correlation with statistical significance at the level (α = 0.05) between stress tolerance and age. The significance of level of patience was (0.487) and the level of stress tolerance was (0.000) (Table 8).

**TABLE 8:** Pearson’s correlation coefficient between patience, stress tolerance, and age.

	Age	
	Pearson correlation coefficient	Level of significance
Patience	0.066	0.487
Stress tolerance	-0.355	0.000

## Discussion

The present results revealed that female heath care workers in Bethlehem Governorate are more susceptible to stress and fear from COVID-19 than males. This may be attributed to the fact that male workers have greater patience than female workers. This finding is in agreement with Panchal et al. (2020), who showed that female health workers’ mental health was negatively impacted because of stress and anxiety form contracting the virus than male workers.

Female workers/women tend to have greater anxiety and fear from the coronavirus than men; this may be due to the fact that men have more patience than women. This is consistent with the Panchal et al. (2020) which showed that women show negative mental health effects due to anxiety and stress from the coronavirus compared to men. This also agrees with van Roekel et al. (2021) and Gramaglia et al. (2021) which revealed that female healthcare workers in direct contact with COVID-19 patients report significantly more sleep problems and physical exhaustion, than male healthcare workers who do not treat COVID-19 patients.

According to WHO reports (World Health Organization, 2022), depression, anxiety, and stress have a greater impact on female health workers than male workers. It is likely that the severity of reaction and its high frequency greatly affect an individual’s ability to control themselves. This may lead to a constant sense of pessimism, anxiety, and stress in one’s life. This may be related to the fact that men are gifted with a higher degree of patience than women (Abdel Hamid, 2020).

The results also showed that patience is higher for single/unmarried workers. This may be attributed to the fact that a single person has no dependents (wife or children) whose wellbeing or health he is likely to worry about. This agrees with a recent study conducted by the Danube University in Austria, with more than 1,000 participants (Academy of Health, 2020). The study revealed that crises and tragedies affect human mental health and social relations. This finding leads to the logical that having no social relations might be reason enough to adapt with adverse conditions such as COVID-19 pandemic.

It was also found that despite being infected with the coronavirus, many Palestinian doctors and nurses endured the physical and psychological pressures they were undergoing and were able to stand in the front lines facing the outbreak of the coronavirus, which has claimed the lives of millions of people around the world and has infected a much greater number of people.

Doctors and nurses, “the white army,” as often called, assume great risk in their attempt to save people disease, in addition to the great psychological pressures they are going through as they follow up on the conditions of the disease and their condition, a number of whom died as a result of this virus (Al-Rimawi, 2021).

The results of the present study are consistent with the results of Puto et al. (2022) that showed healthcare workers during the peak of the COVID-19 pandemic coped with stress using both coping strategies focused on the problem and strategies focused on emotions. Moreover, in this review, valuable evidence was found that showed the value and effectiveness of coping mechanisms, mental resilience and social support in maintaining mental health and psychological well-being among healthcare staff during the COVID-19 pandemic. This also agrees with Maraqa et al. (2021) which showed that physicians and nurses had higher willingness to work than others (*p* = 0.004, Adj. OR = 3.5). Lower stress levels and longer professional experience were predictors of more willing to work (*p* = 0.03, Adj. OR = 2.5; *p* = 0.03, Adj. OR = 2.6, respectively).

It was also found that there is no difference in the specific duties of a health care worker because the medical staff (in any care setting) has acquired many skills to face psychological stress as a result of their experience in dealing with many different diseases (including chronic ones), in addition to their daily exposure to a variety of situations that have over the years contributed much to their acquisition of methods of coping with stress. This agrees with the Hussein and Abdel Azim (2006) that the methods of coping with psychological stress vary from one individual to another according to the age, nature of the stressful situation, the cognitive structure of the individual and his personality type.

However, the results of the current study conflict with those of Khanal et al. (2020) and Khamis et al. (2021) which found that doctors and nurses who cared for COVID-19 patients reported higher levels of stress than those who did not. This might be because of the higher workload and the greater risk of direct contact to with patients with COVID-19.

It was also found that there is a significance in the level of stress tolerance in favor of the National Center in regard to bearing stress. Since this center is dedicated only for the corona disease (COVID-19), the rate of transmission of infection to doctors and nurses exceeds the average that in other hospitals and health centers, and this in turn requires more stress tolerance by employees, most particularly when they go home to their families as the risk of transmitting COVID-19 infection is extremely high. Moreover, workers at this center may themselves be infected by the virus. Hence, health care workers are certainly more vulnerable to infection than members of all other sectors of the society. Yet, their role in combatting the epidemic is completely indispensable despite all odds: the greater risk of infection, and the lack of adequate protection against the transmission of infection.

The study findings indicate there was a negative correlation with statistical significance between stress tolerance and age. Nature of stress changes with age, from episodic to chronic, which in turn affects appraisal and coping processes.

An alternative explanation for the age difference in the amount of reported stress may be that older individuals cope in a different way than their younger counterparts. Various theorists have posited changes in coping with instance. Gutmann (1974) suggested that mastery styles shift from active to passive, from youth to midlife, then to “magical” mastery in late life (Aldwin et al., 1996).

The results of the present study are consistent with the results of Aldwin et al. (1996) and van Roekel et al. (2021) regarding healthcare workers’ age, physical exhaustion is more prevalent among healthcare workers who are older than 55 compared to healthcare workers between 36 and 55 years.

Yet these results are inconsistent with Puto et al. (2022) who showed no significant correlations between the intensity of perceived stress and nurses’ age, both in the case of nurses working with SARS-CoV-2-infected patients and those working with non-infected ones.

## Conclusion

The present study contributes to patience literature by showing there is a significant positive effect for patience on stress tolerance and there were statistically significant differences in the level of patience in favor of males and single workers.

Also results revealed that female heath care workers in Bethlehem Governorate are more susceptible to stress and fear from COVID-19 than males. This finding is in agreement with the literature that female health workers’ mental health was negatively impacted because of stress and anxiety form contracting the virus than male workers.

These findings support the need for early interventions aimed to make sure that the medical departments will hold training workshops aimed at developing the skills of female health care workers on how to cope with stress using both coping strategies focused on the problem and strategies focused on emotions.

One of the limitations faced during the course of this study was getting the questionnaires filled during the beginning of the epidemic before any vaccines were available. Because of that, the methodology was a quantitative only approach.

## Data availability statement

The original contributions presented in this study are included in this article/supplementary material, further inquiries can be directed to the corresponding author.

## Ethics statement

The studies involving human participants were reviewed and approved by Palestinian Ministry of Health. The patients/participants provided their written informed consent to participate in this study.

## Author contributions

NA-A contributed to the design and implementation of the research, to the analysis of the results, and to the writing of the manuscript and approved the submitted version.
